# Activation of the LRR Receptor-Like Kinase PSY1R Requires Transphosphorylation of Residues in the Activation Loop

**DOI:** 10.3389/fpls.2017.02005

**Published:** 2017-11-27

**Authors:** Christian B. Oehlenschlæger, Lotte B. A. Gersby, Nagib Ahsan, Jesper T. Pedersen, Astrid Kristensen, Tsvetelina V. Solakova, Jay J. Thelen, Anja T. Fuglsang

**Affiliations:** ^1^Department of Plant and Environmental Sciences, Faculty of Science, University of Copenhagen, Copenhagen, Denmark; ^2^Christopher S. Bond Life Sciences Center, Department of Biochemistry, University of Missouri, Columbia, MO, United States

**Keywords:** receptor kinase, phosphorylation, mass spectrometry, PSY1 peptide, signaling peptides, LRR, activation loop

## Abstract

PSY1R is a leucine-rich repeat (LRR) receptor-like kinase (RLK) previously shown to act as receptor for the plant peptide hormone PSY1 (peptide containing sulfated tyrosine 1) and to regulate cell expansion. PSY1R phosphorylates and thereby regulates the activity of plasma membrane-localized H^+^-ATPases. While this mechanism has been studied in detail, little is known about how PSY1R itself is activated. Here we studied the activation mechanism of PSY1R. We show that full-length PSY1R interacts with members of the SERK co-receptor family *in planta*. We identified seven *in vitro* autophosphorylation sites on serine and threonine residues within the kinase domain of PSY1R using mass spectrometry. We furthermore show that PSY1R autophosphorylation occurs *in trans* and that the initial transphosphorylation takes place within the activation loop at residues Ser951, Thr959, and Thr963. While Thr959 and Thr963 are conserved among other related plant LRR RLKs, Ser951 is unique to PSY1R. Based on homology modeling we propose that phosphorylation of Ser951 stabilize the inactive conformation of PSY1R.

## Introduction

As multicellular organisms, plants rely on a fine-tuned cell-to-cell communication system to coordinate growth responses. At the core of this system are members of the receptor-like kinase (RLK) superfamily. This massive family of plant proteins, which is implicated in both plant development (De Smet et al., 2009; Murphy et al., 2012) and plant innate immunity (Schwessinger and Ronald, 2012; Tang et al., 2017), maintains an appropriate balance between growth and defense. Plant RLKs belong to the RLK/Pelle class of protein kinases, which is composed of over 600 members in Arabidopsis (Gish and Clark, 2011), and are related to the mammalian Receptor Tyrosine Kinases (RTKs) (Shiu and Bleecker, 2001; Belkhadir et al., 2014). Most plant RLKs share the overall architecture of RTKs, with an extracellular ligand-binding domain, a single transmembrane-spanning domain, and a conserved intracellular kinase domain (KD).

Until recently, it was believed that most plant cell-to-cell communication was mediated by small lipophilic compounds, such as phytohormones and steroids, only. However, plant peptide hormones, which are expressed as precursor peptides (pre-pro-peptides) and undergo post-translational modification followed by secretion via the secretory pathway, are emerging as important players in this process (Matsubayashi, 2011; Murphy et al., 2012). Plant peptide containing sulfated tyrosine 1 (PSY1; Amano et al., 2007) and phytosulfokine (PSK; Matsubayashi and Sakagami, 1996) are plant peptide hormones responsible for cell elongation activity in the elongation/differentiation zone of the root (Sauter et al., 2009; Matsubayashi et al., 2010) and hypocotyl (Stührwohldt et al., 2011; Fuglsang et al., 2014). The receptors for PSY1 and PSK, termed PSY1R and PSKR1 and PSKR2, respectively, form a small family of redundant RLKs (Matsubayashi et al., 2002; Amano et al., 2007) that are members of the leucine-rich repeat (LRR) RLK subclass of plant RLKs, which is encoded by 216 genes in Arabidopsis. The downstream signaling events of PSY1R and PSKR have not been fully characterized; however, PSKR contains a guanylate cyclase catalytic center in its KD, which enables signaling through the formation of the secondary messenger cyclic guanosine monophosphate (cGMP) (Kwezi et al., 2011). PSY1R was recently shown to regulate cell expansion by phosphorylating the plant plasma membrane H^+^-ATPase (AHA2) and thereby promoting proton pumping (Fuglsang et al., 2014). Proton pumping is observed upon addition of PSY1 peptide and is dependent on the presence of PSY1R (Fuglsang et al., 2014). Interestingly, the growth-promoting effects of PSK do not require extracellular acidification by plasma membrane H^+^-ATPases (Stührwohldt et al., 2011) and PSKR1 does not interact with AHA2 in a Bimolecular Fluorescence Complementation (BiFC) assay (Fuglsang et al., 2014), but the two proteins do co-localize in the plasma membrane (Ladwig et al., 2015).

The plant RLK activation mechanism differs from the activation mechanism of mammalian RTKs. In the classical RTK activation known from the mammalian field, ligand-induced RTK homodimerization brings the intracellular KDs into proximity, which thereby allows for intermolecular transphosphorylation and concomitant activation of the KDs and downstream signaling (Lemmon and Schlessinger, 2010). Transphosphorylation within the activation loop is also a central regulatory element of plant RLKs, as demonstrated in phosphorylation studies (Oh et al., 2000; Karlova et al., 2009) and further supported by crystal structures of plant RLK KDs containing phosphorylated residues (Yan et al., 2012; Bojar et al., 2014). Homodimerization, in some cases ligand-induced, has also been observed for a number of plant RLKs, including the *S*-locus Receptor Kinase (SRK) (Giranton et al., 2000), Brassinosteroid Insensitive 1 (BRI1) (Wang et al., 2005b), Flagellin Sensing 2 (FLS2) (Sun et al., 2012), and the CLV/BAM family (Guo et al., 2010).

However, about a decade ago, an interaction partner of BRI1 was identified and named BRI1-Associated Kinase 1 (BAK1) (Li et al., 2002; Nam and Li, 2002). Sequential transphosphorylation within the BRI1/BAK1 complex was demonstrated, showing that BRI1 is fully activated by BAK1 transphosphorylation within the juxtamembrane and C-terminal domains (Wang et al., 2008). BAK1 is also known as Somatic Embryogenesis Receptor-like Kinase 3 (SERK3) and belongs to a small family of SERK proteins consisting of five members (SERK1–5), each containing five LRRs in the extracellular domain (Hecht et al., 2001). SERK proteins are implicated in a range of diverse processes, including plant cell differentiation, growth, and immunity (Ma et al., 2016).

Additionally it has been found that SERKs acts as co-receptors for a range of LRR-RLKs. Each ligand-binding receptor seems to only interact with a limited number of SERK proteins (Ma et al., 2016). For example, BRI1 is regulated by SERK1, SERK4, and BAK1 (SERK3), whereas PSKR1 is regulated by SERK1, SERK2, and BAK1 (Wang et al., 2015). SERK proteins positively modulate the activity of its interaction partners by transphosphorylation (Schulze et al., 2010).

In this study, we investigated the transactivation mechanism of PSY1R and identified its *in vitro* autophosphorylation sites. By homology modeling to known structures of BRI1 and SIRK1 we propose how these residues are involved in stabilization of the activation loop in the active and inactive state, respectively. We furthermore showed that PSY1R interacted with members of the SERK family *in planta*.

## Materials and Methods

### DNA Cloning

A list of primers and plasmids used in this study can be found in the Supplementary Tables 1 and 2. Full-length *SERK2*, *SERK4*, and *SERK5* cDNAs were amplified from an *Arabidopsis thaliana* Col-0 cDNA preparation. A cDNA clone of SERK1 was kindly provided by Professor S. C. de Vries (University of Wageningen). The PCR fragments were subcloned into Gateway pENTR/D-TOPO vector (Invitrogen Life Technologies). The BAK1 cDNA clone in the pCR8/GW/TOPO vector was obtained from Arabidopsis Biological Resource Center (ABRC) (clone CIW00115). The *SERK*, and *BAK1* genes were cloned into Gateway-compatible BiFC vectors by LR recombination using LR Clonase II Enzyme Mix (Invitrogen Life Technologies) to yield C-terminal fusions to cCFP or nYFP expressed from the 35S promoter. The PSY1R BiFC constructs were used in a previous study (Fuglsang et al., 2014) and the PSY1R K831A mutation, corresponding to the invariant lysine residue present in the protein kinase catalytic domain (Carrera et al., 1993), was generated through QuikChange Site Directed Mutagenesis (Agilent Technologies).

To generate constructs for expression in *E. coli*, the region of *PSY1R* encoding the entire intracellular domain of PSY1R was amplified with gene-specific primers carrying a 5′ CACC overhang for subcloning in the Gateway pENTR/D-TOPO vector (Invitrogen Life Technologies). Mutations and stop codons were introduced through QuikChange Site Directed Mutagenesis. The constructs were transferred into pDEST15 and pDEST17 (Invitrogen Life Technologies) through the LR reaction. All constructs were sequenced by Eurofins MWG Operon.

### Transient Expression in *Nicotiana benthamiana*

Transformed *Agrobacterium tumefaciens* strain C58C1 was grown overnight in liquid YEP medium containing 25 μg/mL gentamicin and 50 μg/mL spectinomycin. Cells were washed and resuspended in infiltration solution (10 mM MgCl_2_, 100 μM acetosyringone), and diluted to an OD_600_ of 0.05, before mixing the transformed cells in the combinations to be tested. *N. benthamiana* leaves were infiltrated with the *A. tumefaciens* mix using a needleless syringe. Fluorescence was monitored approximately 48 h after infiltration.

### Confocal Microscopy

A Leica SP5 confocal laser-scanning microscope with a 20 × 0.7 numerical aperture water-immersion objective was used to examine the lower epidermis of the infiltrated tobacco leaves. The complemented YFP/CFP fluorescence was excited at 448 nm and emission was detected at 515–540 nm. The gain was fixed in all samples to ensure that the emission intensity was comparable. Interaction was tested using both combinations of fusion proteins with similar results.

### Expression and Purification of Recombinant Protein in *E. coli*

GST-tagged proteins were expressed either from the pGEX-4T-1 vector (GE Healthcare Life Sciences) in BL21(DE3) cells or from the pDEST15 vector in BL21-AI cells (Invitrogen Life Technologies). Briefly, LB medium was inoculated with an overnight LB + 100 μg/mL ampicillin culture to a start OD_600_ of 0.1 and grown at 37°C until the OD_600_ reached 0.5–0.7. Expression from the pDEST15 plasmid in BL21-AI cells was induced by the addition of L-(+)-arabinose to a final concentration of 0.2% (w/v) and the culture was incubated at 20°C overnight. Expression from the pGEX-4T-1 vector in BL21(DE3) cells was induced by the addition of 100 μM IPTG and the culture was incubated at 28°C for 3–4 h. In both cases, the cells were harvested at 14,000×*g* for 10 min, washed in cold H_2_O, and pelleted again. The cells were resuspended in P-buffer (50 mM Tris–HCl, 150 mM NaCl, pH 7.5) containing 1 mM PMSF, 0.01% (w/v) DNase I, and 0.01% (w/v) lysozyme and lysed by sonication. The cell debris was collected by centrifugation at 15,000×*g* for 30 min and the lysate was incubated with Glutathione Sepharose 4B (GE Healthcare) for 2 h at 4°C. The resin was washed three times with P-buffer before eluting the protein with 50 mM L-glutathione in P-buffer, adjusted to pH 8.0. His-tagged proteins were expressed from the pDEST17 plasmid in BL21-AI cells as described above. The cells were harvested as described above, resuspended in lysis buffer (50 mM Na-phosphate, 300 mM NaCl, 10 mM imidazole, pH 8.0) containing 1 mM PMSF, 0.01% (w/v) DNase I, and 0.01% (w/v) lysozyme and opened by sonication. The cell lysate was collected as described above and incubated with Ni-NTA agarose (Qiagen) for 2 h at 4°C. The resin was washed three times with wash buffer (as lysis buffer, but containing 60 mM imidazole) before eluting the protein with elution buffer (as lysis buffer, but containing 250 mM imidazole).

### Phosphorylation Assays

In a radiometric assay, purified kinase was incubated for 30 min at 30°C in kinase assay buffer (50 mM HEPES-NaOH pH 7.2, 150 mM NaCl, 10 mM MgCl_2_, 1 mM DTT) with 50 μM ATP and 5 μCi γ-^32^P-ATP (PerkinElmer) in a volume of 50 μL. The reaction was stopped by the addition of 10 μL 100% (w/v) TCA. The TCA-precipitated protein was dissolved in twofold concentrated Laemmli buffer and proteins were separated on a 10% SDS-PAGE gel. After the gel was stained and dried, it was exposed to a phosphor screen for 3 days and phosphorylation was detected using a Storm 860 scanner (Molecular Dynamics).

For detection of phosphorylated proteins with phosphoamino acid-specific antibodies, purified kinase was incubated for 1 h at 30°C in kinase assay buffer with 500 μM ATP. As above, the reaction was stopped by TCA precipitation and proteins were separated by SDS-PAGE. The protein bands were blotted onto a nitrocellulose membrane and the membrane was blocked with 3% (w/v) BSA. Rabbit polyclonal phosphoserine (Invitrogen Life Technologies, 61-8100, 1:200) and phosphothreonine (Invitrogen Life Technologies, 71-8200, 1:1000) antibodies were used as primary antibodies and polyclonal goat anti-rabbit alkaline phosphatase-coupled immunoglobulin (DAKO, D0487, 1:2000) were used as secondary antibody. The immunoblot was developed using NBT/BCIP substrate (Promega, S3771).

### Homology Model of PSYR1 Kinase Domain

Modeller v9.19 software^1^ (Sali and Blundell, 1993) was used to generate the homology models with maize SIRK1 KD (PDB ID: SIRK1) and BRI1 KD (PDB ID: 5LPV) as templates. The templates were alignment with the KD of PSY1R (residue 781–1079) individually and as a multiple alignment with both templates. Models were initially evaluated using discrete optimized protein energy (DOPE) score and using energy plots that show problematic regions. The generated models were further evaluated and energy refined using the SAVES^2^ and GALAXY (Heo et al., 2013) server, respectively. Final models were visualized using PyMOL (The PyMOL Molecular Graphics System, Version 1.8 Schrödinger, LLC) and the inhibitor AMP-PNP and Mg^2+^ were modeled into the homology models to visualize the catalytic ATP binding site. The energy plots were visualized using Gnuplot 5.0.

### MS/MS Detection of Phosphosites

Auto- and transphosphorylated sites of the intracellular domain of PSY1R were detected using a LTQ Orbitrap XL ETD mass spectrometer (Thermo Fisher Scientific, San Jose, CA, United States). Full-length recombinant proteins were digested in-solution and/or in-gel with sequencing grade trypsin (Promega, Madison, WI, United States). Freeze-dried tryptic peptides were dissolved by adding 40 μL of 0.1% formic acid and subjected to MS analysis. Ten microliters of each sample was resolved using a Finnigan Surveyor liquid chromatography system interfaced with the mass spectrometer. Tryptic peptides were fragmented using either collision-induced dissociation (CID) or “decision tree” methods that utilize both CID and electron-transfer dissociation (Swaney et al., 2008).

The MS RAW files were searched against the TAIR10 database combined with a decoy database containing the randomized sequences of the original database. The search parameters, described in detail previously (Ahsan et al., 2013), were briefly as follows: the mass type, average precursor plus fragment; dynamic modifications, phosphorylation of Ser/Thr/Tyr (+79.9799 Da) and oxidation of Met (+15.9994 Da); and the static modification, Cys-carboxyamidomethylation.

Identification data were evaluated using the XCorr function of SEQUEST, and phosphorylation-site localization was accomplished using phosphoRS (Proteome Discoverer, v. 1.0.3, Thermo Fisher Scientific). The XCorr values for each charge state were set to default, and no decoy hits were allowed. Peptide mass deviation was 10 ppm and two peptides/protein were used to further filter the data. Phosphopeptides with a pRS score of ≥50 and a pRS site probability of ≥50% were considered as high-confidence phosphosites. For final validation, each spectrum was inspected manually and accepted only when the phosphopeptide had the highest pRS site probability, pRS score, XCorr value, and site-determining fragment ions allowed for unambiguous localization of the phosphorylation site.

## Results

### Identification of PSY1R *in Vitro* Autophosphorylation Sites

**FIGURE 1:**
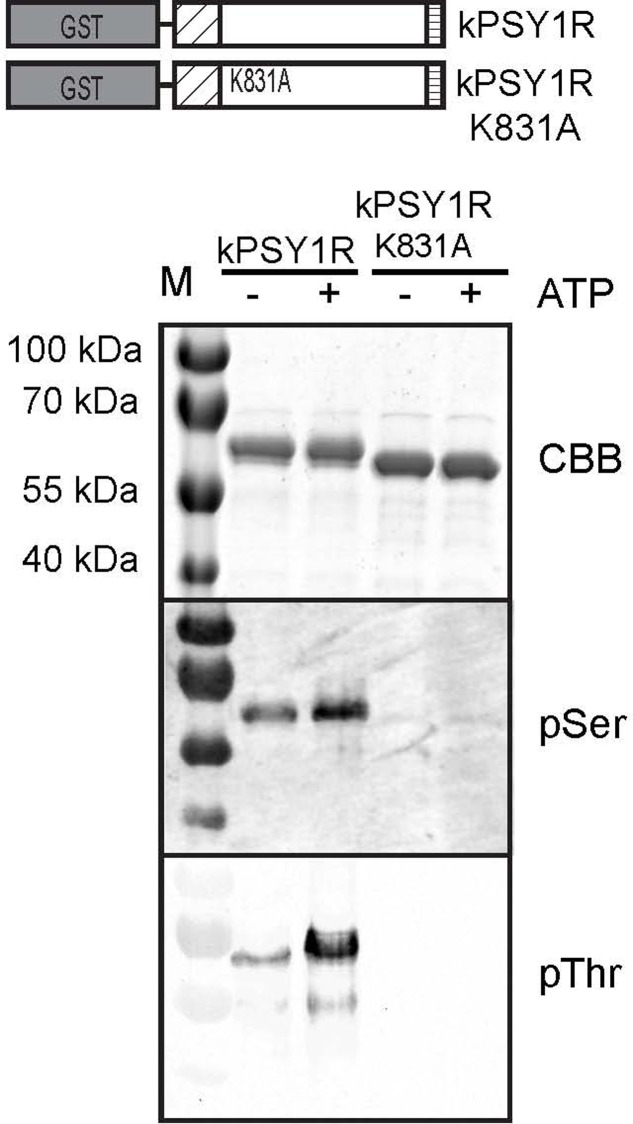
PSY1R autophosphorylates on serine and threonine residues. The intracellular domain of PSY1R, was expressed in *E. coli* with a GST tag at the N-terminal end (kPSY1R). The kPSY1R K831A variant harbors the K831A mutation, which renders the kinase inactive. GST-purified kPSY1R or kPSY1RK831A was incubated in the presence or absence of ATP in an *in vitro* kinase assay and phosphorylation of the proteins was probed with phosphoamino acid-specific antibodies.

We set out to investigate the autophosphorylation mechanism of PSY1R. The cytosolic KD of PSY1R (residues 741–1095; kPSY1R) were expressed in *E. coli* as GST-fused protein (**Figure 1**). An inactive variant of the kinase were generated by mutating the lysine residue in the catalytic domain to an alanine (kPSY1R K831A). Recombinant kPSY1R were incubated in an *in vitro* kinase assay in the absence or presence of ATP and phosphorylated amino acids were detected with phosphoamino acid-specific antibodies. As seen in **Figure 1**, phosphoserine (pSer) and phosphothreonine (pThr) antibodies reacted with the active kPSY1R protein, but weakly or not at all with the inactive kPSY1R K831A. This implies that PSY1R autophosphorylates on Ser and Thr residues. The addition of ATP to the kinase reaction assay increased the phosphorylation level especially of Thr residues. The presence of phosphorylated residues in samples without ATP indicates the kinase is active and autophosphorylated when expressed in *E. coli*. The detected phosphorylation of kPSY1R is, however, true autophosphorylation, since phosphorylation of the inactive kPSY1R K831A was nearly undetectable.

**FIGURE 2:**
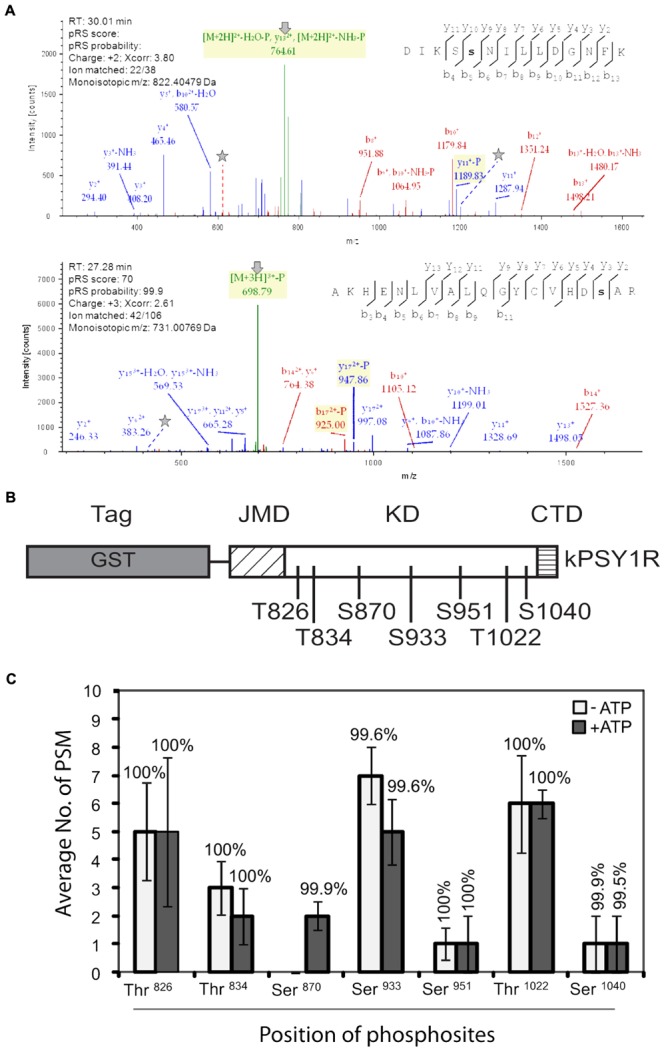
Mapping of autophosphorylation sites in recombinant kPSY1R. **(A)** Upper panel: The MS/MS spectrum of the doubly charged phosphopeptide DIKSsNILLDGNFK provides evidence of phosphorylation of Ser933, which is found in the Ser/Thr protein kinase active site signature. Lower panel: The MS/MS spectrum of the triple charged peptide AKHENLVALQGYCVHDsAR provides evidence for phosphorylation of Ser870 within the kinase domain. N-terminal and C-terminal peptide sequence ions are indicated with b_i_ and y_i_, respectively. In the spectra, lines and stars indicate the neutral loss of the phosphate on fragments or precursor ions and the phosphorylated residues, respectively. **(B)** Linear domain structure of the intracellular domain of PSY1R revealing the localization of the seven autophosphorylation sites in the kinase domain identified by MS. **(C)** Quantitative MS analysis of the autophosphorylation sites. Spectral count data (peptide spectral matches, PSM) are the means of four replicates. Percentages above the data bars indicate the pRS site probability for each amino acid. Solid and empty bars represent the presence and absence of ATP, respectively. Error bars represent means ± SE of four independent replicates.

We then mapped the autophosphorylation sites of the intracellular domain of PSY1R. We incubated kPSY1R in the absence or presence of ATP to allow autophosphorylation. Afterward the proteins were digested with trypsin. The tryptic peptides were analyzed by LTQ-Orbitrap high-resolution mass spectrometry (MS/MS) to map phosphorylation events. Examples of MS/MS spectra for two of the determined autophosphorylation sites are shown in **Figure 2A**. These spectra presented evidence for phosphorylation of the peptides AKHENLVALQGYCVHDsAR and DIKSsNILLDGNFK (lowercase letters denote phosphorylated residues), which contain the Ser870 and Ser933 phosphosites, respectively. We unambiguously identified seven total autophosphorylation sites (**Figure 2B**), including three threonine and four serine residues, each of which were located within the KD. Detailed information on the identified phosphopeptides is listed in **Table 1**. The PSY1R intracellular domain contains 12 possible phosphorylation targets within the juxtamembrane domain (JMD) (8 Ser, 2 Thr, and 2 Tyr), 35 possible targets within the KD (13 Ser, 11 Thr, and 11 Tyr), and 1 possible target within the C-terminal domain (1 Thr). However, autophosphorylation of JMD or C-terminal domain residues was not detected in our MS analysis. Spectral count analyses revealed that the addition of ATP during the *in vitro* kinase assay enhanced autophosphorylation of Ser870, whereas the remaining six phosphosites were also identified in the control lacking ATP (**Figure 2C**).

**Table 1:** Mass spectrometry data on the peptides carrying the PSY1R *in vitro* autophosphorylation sites.

Phosphosite	Phosphopeptide identified	Δ*M* (ppm)	pRS score^a^	pRS site probability^b^	XCorr	Charge	*m/z* (Da)	Ions matched
Thr826	ATLDNGpTKLAVK	1.63	80	T(2): 0.0; T(7): 100.0	3.76	2	1310.6694	23/32
Thr834	KLpTGDYGMMEK	0.87	163	T(3):100.0; Y(6): 0.0	3.97	2	1352.5615	26/29
Ser870	AKHENLVALQGYCVHDpSAR	1.36	70	Y(12): 0.1; S(17): 99.9	2.61	3	2191.0085	42/106
Ser933	DIKSpSNILLDGNFK	0.58	127	S(4): 95.3; S(5): 4.7	4.41	2	1643.8031	28/38
Ser951	AYVADFGLpSR	1.03	150	Y(2): 0.0; S(9): 100.0	3.64	2	1178.5230	25/26
Thr1022	ELVAWVHpTMKR	1.59	113	T(8): 100.0	2.71	2	1449.7050	24/29
Ser1040	DGKPEEVFDTLLREpSGNEEAMLR	0.02	70	T(10): 0.1; S(15): 99.9	3.09	3	2715.2332	47/130


*^a^pRS score. This peptide score is based on the cumulative binomial probability that the observed match is a random event. The value of the pRS score strongly depends on the data scored, but usually scores of above 50 give good evidence for a good PSM. ^b^pRS site probabilities. For each phosphorylation site this is an estimation of the probability for the respective site being truly phosphorylated.*

### Autophosphorylation of PSY1R Occurs *in Trans*

**FIGURE 3:**
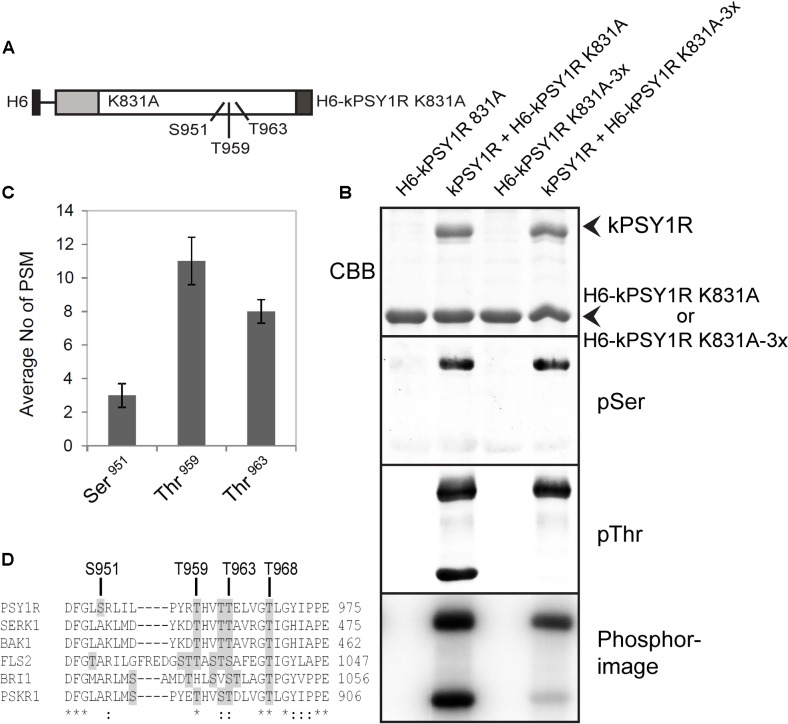
First phase transphosphorylation sites of kPSY1R. **(A)** Linear domain structure of the intracellular domain of the H_6_-kPSY1R K831A inactive mutant revealing the localization of the three transphosphorylation sites identified by MS. Juxtamembrane domain (JMD) is indicated by gray and C-terminal domain by dark gray shading. **(B)** The GST-tagged kPSY1R is able to transphosphorylate the inactive His-tagged kPSY1R K831A in an *in vitro* kinase assay with radiolabeled ATP. Transphosphorylation of H_6_-kPSY1R K831A studied using a combination of phosphoserine and phosphothreonine antibodies and radiolabeled ATP. Mutation of the three transphosphorylation sites (3x = S951A, T959A, and T963A) identified by MS prevents transphosphorylation on threonine residues while overall transphosphorylation is reduced markedly as detected with radiolabeled ATP. **(C)** Average phosphopeptide spectral matches (PSM) identified for each phosphosite. The data represent the means ± SE of two independent replicates. **(D)** Amino acid sequence alignment of the kinase activation loop of selected plant LRR receptor-like kinases. The activation loop begins at the DFG motif and ends with the A/PPE motif. Serine and threonine residues are shaded in gray. The location of the transphosphorylated sites in PSY1R are indicated above the alignment. Asterisks denote conserved residues, while colons denote partially conserved or similar residues at the indicated position.

Since the full-length LRR RLK PSY1R protein was previously shown to form homodimers, we were prompted to ask whether the intracellular kinase activation mechanism involved inter- or intramolecular phosphorylation, i.e., *trans*- or *cis*-phosphorylation. To investigate this, we constructed a hexahistidine-tagged inactive variant of the intracellular domain of PSY1R (H_6_-kPSY1R K831A, **Figure 3A**) and incubated it with the active kPSY1R protein. Interestingly, the inactive intracellular domain was phosphorylated in the presence of the active kPSY1R, but not when it was incubated alone (**Figure 3B**, phosphorimage), which demonstrates that *in vitro* autophosphorylation of the intracellular domain occurs *in trans*. Transphosphorylation of the inactive H_6_-kPSY1R K831A by the active kPSY1R was detected when using the pThr antibody, but not with the pSer antibody (**Figure 3B**).

### First Phase Transphosphorylation Sites Include Residues within the Kinase Activation Loop

To examine the transphosphorylation mechanism of kPSY1R in detail, we mapped the transphosphorylation sites of kPSY1R by MS. We incubated the inactive H_6_-kPSY1R K831A with ATP in the presence or absence of active kPSY1R (GST-tagged) to allow for transphosphorylation. MS analysis identified three phosphorylated sites, Ser951, Thr959, and Thr963, within the KD of H_6_-kPSY1R K831A after incubation with kPSY1R (**Figure 3B**). No phosphorylated sites were detected in H_6_-kPSY1R K831A incubated without kPSY1R. Detailed data for the three phosphopeptides AYVADFGLsR, tHVTTELVGTLGYIPPEYGQAWVATLR, and LILPYRTHVTtELVGTLGYIPPEYGQAWVATLR harboring Ser951, Thr959, and Thr963, respectively, are provided in **Table 2**. Interestingly, all three transphosphorylation sites are located within the kinase activation loop, which was previously shown to carry phosphosites important for catalytic kinase activity in other kinases (Huse and Kuriyan, 2002). Spectral count analysis demonstrated that the phosphorylation level of Thr959 and Thr963 was higher than that of Ser951 (**Figure 3C**). This explains why transphosphorylated Ser951 is not detected by the pSer antibody (**Figure 3B**), as this residue has low phosphorylation levels compared to Thr959 and Thr963. Alignment of the activation loops from a number of plant LRR RLKs, showed that residues corresponding to Thr959 and Thr963 are conserved within in the activation loop whereas the Ser951 residue is unique for PSY1R (**Figure 3D**).

**Table 2:** Mass spectrometry data on the peptides carrying the PSY1R *in vitro* transphosphorylation sites.

Phosphosite	Phosphopeptide identified	Δ*M* (ppm)	pRS score^a^	pRS site probability^b^	XCorr	Charge	*m/z* (Da)	Ions matched
Ser951	AYVADFGLpSR	3.22	138	Y(2): 0.0; S(9): 100.0	2.69	2	589.7676	22/26
Thr959	pTHVTTELVGTLGYIPPEYGQ-AWVATLR	3.13	90	T(1): 68.6; T(5): 10.5	4.98	3	1018.1807	63/208
Thr963	LILPYRTHVTpTELVGTLGYIP-PEYGQAWVATLR	3.82	53	T(7): 5.0; T(11): 40.0	4.22	4	952.7563	65/285


*^a^pRS score. This peptide score is based on the cumulative binomial probability that the observed match is a random event. The value of the pRS score strongly depends on the data scored, but usually scores of above 50 give good evidence for a good PSM. ^b^pRS site probabilities. For each phosphorylation site this is an estimation of the probability for the respective site being truly phosphorylated.*

To verify the transphosphorylation sites identified by MS, we created a new construct (H_6_-kPSY1R K831A-3x) in which all three proposed transphosphorylation sites (Ser951, Thr959, and Thr963) were mutated to alanine. We then asked whether the active kPSY1R could transphosphorylate H_6_-kPSY1R K831A-3x. As shown in **Figure 3B**, transphosphorylation of threonine residues was completely abolished in H_6_-kPSY1R K831A-3x. This indicates that Thr959 and Thr963 are exclusive first-phase transphosphorylation sites.

### Residue Thr968 Is Indispensable for kPSY1R Kinase Activity, While Ser951 Is a Possible Negative Regulatory Phosphorylation Site

**FIGURE 4:**
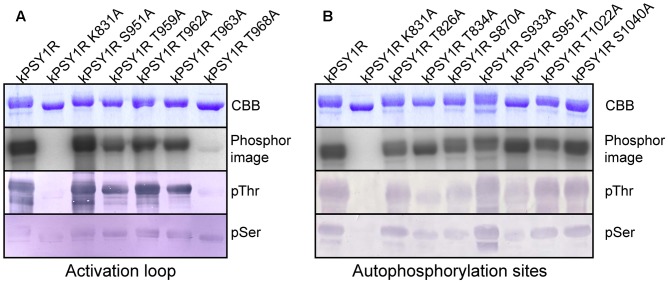
Identification of possible phosphorylation sites that regulate PSY1R kinase activity. The autophosphorylation activity of different mutants of the intracellular domain of PSY1R was evaluated in an *in vitro* kinase assay with ^32^P-labeled ATP. **(A)** Serine and threonine residues within the activation loop were mutated individually to alanine and the autophosphorylation activity of each mutant was compared to that of the wild type (kPSY1R) and inactive kinase mutant (kPSY1R K831A). **(B)** Serine and threonine residues identified as autophosphorylation sites by MS analysis were mutated individually to alanine and the autophosphorylation activity of each mutant was compared to that of the wild type (kPSY1R) and inactive K831A mutant (kPSY1R K831A).

To evaluate the *in vitro* function of each possible autophosphorylation site on the activity of kPSY1R, we generated a series of point mutations. Each of the autophosphorylation sites identified by MS as well as all possible phosphorylation sites within the activation loop were mutated individually to alanine residues in wild-type kPSY1R, thereby preventing phosphorylation at the corresponding residue. As shown in **Figure 4A**, only mutation of residue T968 in the activation loop affected the autophosphorylation severely, resulting in a nearly inactive kinase. By contrast, mutation of S951 led to an increased autophosphorylation level, suggesting that phosphorylation of S951 negatively regulates kPSY1R activity (**Figure 4**).

**FIGURE 5:**
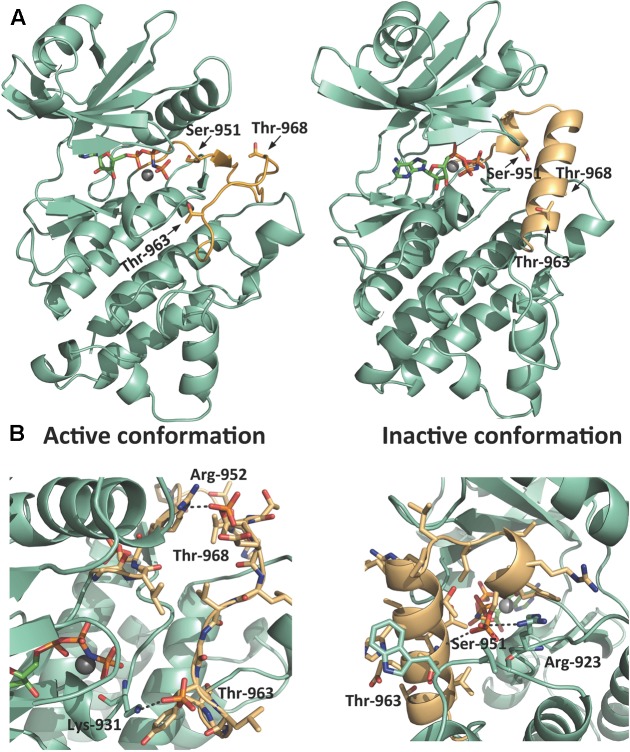
Homology model of PSY1R kinase domain. A homology model of PSY1R kinase domain in both the active (left) and inactive (right) conformation. **(A)** The activation loop (light orange) starting from the conserved DFG motif contains multiple phosphorylation sites. Whereas the overall fold of the rest of the kinase domain (green cyan) is conserved between the two activation states, the active conformation of the activation loop is less structured compared to the inactive kinase. **(B)** Phosphorylation of the two threonine residues give rise to possible salt bridges between the phosphate groups and Lys-931 and Arg-952 that may stabilize the active kinase (left figure). Phosphorylation of Ser-951 stabilizes in contrast the inactive conformation (right figure) through a hydrogen bond between the phosphate and the backbone of Thr-963 and a possible salt bridge to Arg-923. The active site in all models is marked with the ATP analog AMP-PNP and Mg^2+^ modeled from the template structures (PDB-id 5LPV and 5UV4).

To visualize and to identify a possible structural explanation for this positive and negative regulation by Ser/Thr phosphorylation we used Modeller 9.19 to predict a homology model of PSY1R KD (**Figure 5**). PSY1R was modeled in the active conformation using the crystal structure of phosphorylated BRI1 KD as template (Bojar et al., 2014). To predict PSY1R in the inactive conformation the recently published crystal structure of the maize SIRK1 KD was used as template (Aquino et al., 2017). However, as the sequence identity between the KDs of SIRK1 and PSY1R is relatively low (34%) and the energy plot (Supplementary Figure 1) indicated several problematic regions the crystal structure of BRI1 was included as template. The model based on the two templates showed a significantly better energy plot than models build on SIRK1 alone as seen in Supplementary Figure 1. The inactive conformation of LRR-RLKs is characterized by a structured activation loop (**Figure 5A**) (Aquino et al., 2017). As seen in **Figure 5A**, the homology model based on the two templates shows a much more structured activation loop than the model based on BRI1 alone indicating that the model is in the inactive conformation. We next added a phosphate group to Ser-951, Thr-963, and Thr-968 to see whether phosphorylation stabilizes either the active or inactive conformation. As seen in **Figure 5B**, addition of phosphate to the two threonine residues results in two possible salt bridges that can stabilize the active conformation whereas phosphorylation in the inactive conformation did not give rise to any stabilizing interactions (Supplementary Figure 2). In contrast, phosphorylation of Ser-951 seems to stabilize the inactive conformation only as the phosphate group can interact with both the backbone of Thr-963 and Arg-923. These observations support that phosphorylation of Thr-968 leads to increased autophosphorylation whereas Ser-951 seems to be a negative regulatory phosphorylation site.

### kPSY1R Interacts with Members of the SERK Family

**FIGURE 6:**
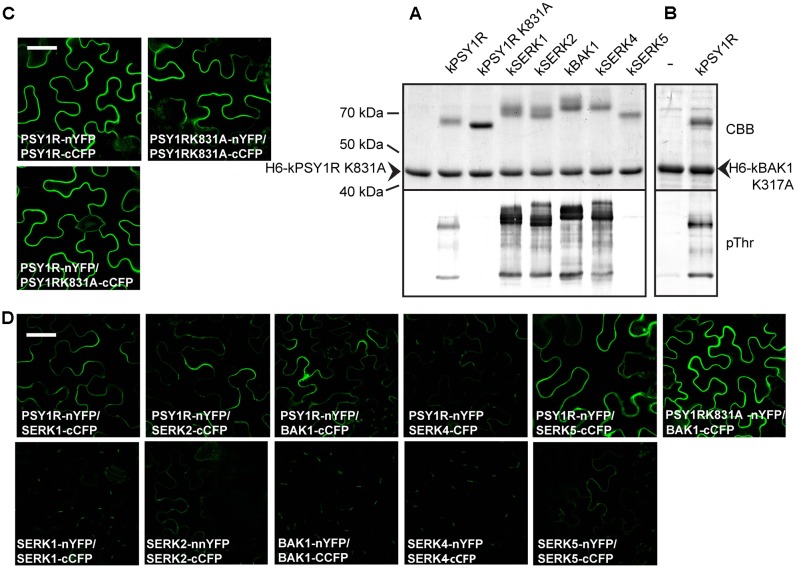
Members from the SERK family transphosphorylate kPSY1R. **(A)** The GST-tagged kPSY1R is able to transphosphorylate the inactive His-tagged kPSY1R K831A in an *in vitro* kinase assay with radiolabeled ATP. Upper panel: CBB stained; Lower panel: Phosphor image. **(B)** Transphosphorylation between intracellular domains of PSY1R and members of the SERK family. SERK1, 2, and 4 and BAK1 transphosphorylate kPSY1R K831A and kPSY1R transphosphorylates kBAK1 K317A *in vitro*. SERK5 exhibits low autophosphorylation activity and does not transphosphorylate kPSY1R. Upper panel: CBB staining; Lower panel: Anti-phosphothreonine blot. **(C,D)** BiFC analysis of PSY1R and SERK interactions in *N. benthamiana* leaves. The proteins are fused to either the N-terminal part of YFP (nYFP) or the C-terminal part of CFP (cCFP) as indicated on the figure. All pictures are taken with the same magnification, scale bar = 50 μm. **(C)** PSY1R and PSY1R K831A form homodimers. **(D)** PSY1R interacts with members of the SERK family to varying degrees. Interaction between PSY1R and BAK1 is not dependent on PSY1R kinase activity. The SERK proteins do not form or only form weak homodimers in this assay. All pictures are taken with the same magnification, scale bar = 50 μm.

BAK1 and other members of the SERK family were previously shown to act as co-receptors for LRR-RLKs such as BRI1 and FLS2. To test whether SERK proteins could regulate PSY1R activity through phosphorylation, we created GST-tagged constructs of the intracellular domain of the SERK proteins (kSERK and kBAK) and tested whether these were able to transphosphorylate the inactive H6-kPSY1R K831A protein. In this case, we used the pThr antibody to detect transphosphorylation. As shown in **Figure 6A**, the intracellular domains of SERK1, SERK2, BAK1, and SERK4 were all able to transphosphorylate H_6_-kPSY1R K831A, whereas kSERK5 exhibited weak autophosphorylation activity and did not transphosphorylate H_6_-kPSY1R K831A. In the *Col*-0 accession, SERK5 harbors an R401L mutation in the otherwise conserved HRD motif of the KD, which could explain the lack of autophosphorylation activity observed in our assay. To test the ability of kPSY1R to transphosphorylate a SERK protein, we constructed an inactive H_6_-tagged version of the intracellular domain of BAK1, H_6_-mBAK1 K317A. As seen in **Figure 6B**, kPSY1R was able to transphosphorylate H_6_-BAK1 K317A. Taken together, these results suggest that one or more of the SERK proteins act as co-receptors for PSY1R via a trans-activation mechanism.

### PSY1R Can Form Homo- and Heterodimers *in Planta*

Using a BiFC assay, we evaluated the ability of full-length PSY1R and PSY1R mutants to dimerize *in planta*. As seen in **Figure 6C**, full-length PSY1R forms homodimers *in planta*, as we reported previously (Fuglsang et al., 2014). PSYR1 protein, harboring a K831A mutation in the KD (*psy1r*K831A) resulting in loss of kinase activity, also forms homodimers *in planta* (**Figure 6C**).

We further investigated whether PSY1R also forms complexes with SERK proteins. As shown in **Figure 6D**, PSY1R interacts with SERK5, even though SERK5 seems inactive, and to a lesser extent with BAK1, SERK1, and SERK2, whereas PSY1R barely interacted with SERK4 in the employed BiFC assay. All the interactions take place at the plasma membrane as expected. We did not detect homodimerization of any of the SERK members. Furthermore, PSY1R K831A forms a strong complex with BAK1.

## Discussion

Taken together, we identified three residues within the activation loop of PSY1R, which are part of the first phase transphosphorylation mechanism. By homology modeling to known structures of BRI1 and SIRK1 we demonstrated how these residues are involved in stabilization of the activation loop in the active and inactive state, respectively. Finally we demonstrated that PSY1R interacts with co-receptors from the SERK family and that kPSY1R are target for SERK kinases.

Mapping of the PSY1R autophosphorylation sites by MS revealed a total of seven autophosphorylation sites, four serine and three threonine residues, all located within the KD, including a single phosphosite (Ser951) within the activation loop. It is surprising that we did not detect any phosphorylation sites within the JMD previously reported to be important for the function of plant RLKs (Yoshida and Parniske, 2005; Xu et al., 2006; Petutschnig et al., 2010; Meyer et al., 2013). The *in vivo* phosphorylation site database PhosPhAt 4.0 (Durek et al., 2010) contains two PSY1R phosphorylation sites, Ser8 and Ser10, which are probably not authentic PSY1R autophosphorylation sites, as they are located within the predicted signal sequence.

Here we have demonstrated that PSY1R first-phase transphosphorylation occurs within the activation loop, specifically at residues Ser951, Thr959, and Thr963. Phosphorylation of Thr959 and Thr963 were only found in the transphosphorylation experiments and not in the initial mapping of phosphosites. This indicates that these sites are only transiently phosphorylated during the catalytic cycle and therefore caught in the inactive mutant; alternatively a full-length receptor protein is required in order to maintain the conformation with these sites phosphorylated. As seen from an alignment of the activation loops of a number of plant LRR RLKs (**Figure 3D**), the Ser951 residue is not conserved among the aligned RLKs and is unique for PSY1R. Based on homology modeling we propose that the phosphorylated Ser951 stabilizes the inactive conformation of PSY1R. In addition to Ser951, PSY1R contains four threonine residues, Thr959, Thr962, Thr963, and Thr968, which are conserved among the plant RLKs shown here. The residues within the activation loop of plant RLKs are key phosphorylation sites that are important for kinase activity (Oh et al., 2000; Shah et al., 2001; Wang et al., 2005a; Karlova et al., 2009). In the crystal structure of the BAK1 KD, all four threonine residues within the activation loop are phosphorylated and are involved in electrostatic interactions that maintain the conformation of the activation and catalytic loop (Yan et al., 2012). Structurally, the most important residue in BAK1 is Thr450, as it plays a role analogous to the single threonine residue (Thr197) within the activation loop of the mammalian kinase Protein Kinase A (Johnson et al., 1996). Interestingly, we found that the residue in PSY1R that corresponds to BAK1 Thr450, the Thr963 residue, was one of the initial transphosphorylation sites. Based on homology modeling, we speculate that the phosphorylated PSY1R Thr963 residue plays an important role in maintaining the active conformation of an activation loop.

Plant RLKs are categorized into two groups based on whether they are activated through intermolecular *trans*-phosphorylation or intramolecular *cis*-phosphorylation. Members of the former group are able to transphosphorylate an inactive version of the KD and show second order kinetics with respect to the kinase concentration and include BRI1 (Wang et al., 2005b), CLV1 (Williams et al., 1997), SERK1 (Shah et al., 2001), and HAESA (Horn and Walker, 1994; Taylor et al., 2013). Members of the latter group do not transphosphorylate an inactive KD and exhibit first order kinetics with respect to kinase concentration and include AtACR4 (Meyer et al., 2011), CrRLK1 (Schulze-Muth et al., 1996), and Xa21 (Liu et al., 2002). In our study, we observed that the KD of PSY1R transphosphorylated the inactive mutant PSY1R K831A. Therefore, we speculate that transphosphorylation is an important event in PSY1R signaling analogous to the proposed activation mechanism of BRI1 (Wang et al., 2008). BRI1 is first partially activated through transphosphorylation within the activation loop. The partially activated BRI1 receptor interacts with and phosphorylates BAK1, which in turn phosphorylates BRI1 on residues within the JMD and C-terminal domain, which fully activates BRI1 (Oh et al., 2014).

It is tempting to speculate that PSY1R is fully activated by SERK phosphorylation, analogous to BRI1. The requirement for a co-receptor would also explain the lack of detected phosphosites in the PSY1R JMD, as this would require the presence of a co-receptor in the *in vitro* assay applied for MS studies. In the crystal structure of the closely related PSK receptor PSKR1, a co-receptor is not required for ligand binding but the potential involvement of a co-receptor for the complex activation was suggested and it was found that PSK induces the heterodimerization of PSKR1 with SERK1, SERK2, and SERK3 *in planta* (Wang et al., 2015).

Additionally, we demonstrated *in planta* interactions between PSY1R and members of the SERK family, which act as co-receptors and regulate distinct signaling pathways (Chinchilla et al., 2009; Li, 2010). SERK proteins are implicated in controlling the balance between growth and defense, as they regulate the activity of receptors involved in plant development and in plant immunity. Our results suggest that SERK proteins also regulate PSY1R signaling. Based on BiFC analyses, we observed differences in the fluorescence intensity of the signal when testing for interactions between PSY1R and different SERK members. This might indicate differences in the stability of the interaction. Interestingly we observe a very strong interaction between the inactive PSY1R K831A mutant and BAK1 as well as between the inactive SERK5 and PSY1R. This shows that protein kinase activity is not required for the interaction, as previously observed within this family (Karlova et al., 2009). Also it could indicate that the interaction between the two kinases is less transient, compared to a situation with two active kinases, which therefore results in formation of more fluorescent molecules. Future studies will reveal whether the interactions between PSY1R and members of the SERK family are of functional relevance *in vivo*.

## Conclusion

Our study has revealed details of the regulation of the kinase activity of PSY1R. Future studies will reveal the *in vivo* function of the identified autophosphorylation sites and how the interaction with the SERK co-receptor proteins fine-tunes PSY1R signaling.

## Author Contributions

CO and AF designed the study. CO, LG, and TS performed the kinase phosphorylation analysis. CO and AK performed the BiFC analysis. NA performed the MS analysis. JP performed the structural analysis. CO, JT, and AF supervised the project.CO and AF wrote the manuscript. All authors reviewed the results and approved the final version of the manuscript.

## Conflict of Interest Statement

The authors declare that the research was conducted in the absence of any commercial or financial relationships that could be construed as a potential conflict of interest.

## Footnotes

1 http://www.salilab.org

2 https://services.mbi.ucla.edu/SAVES/
