# Supplementary material for: Activation of the LRR Receptor-Like Kinase PSY1R Requires Transphosphorylation of Residues in the Activation Loop
Source: Front Plant Sci. 2017 Nov 27;8:2005. doi: 10.3389/fpls.2017.02005 (PMC5712095; doi:10.3389/fpls.2017.02005)
Supplement: Supplementary file 1 [file Table_1.DOCX]

Supplementary Material

Activation of the LRR Receptor-Like Kinase PSY1R requires transphosphorylation of residues in the activation loop

**Christian B. Oehlenschlæger^1^, Lotte B. A. Gersby^1^, Nagib Ahsan^2,3^, Jesper T. Pedersen^1^, Astrid Kristensen^1^, Tsvetelina V. Solakova^1^, Jay J. Thelen^2^, and Anja T. Fuglsang^1*^**

*** Correspondence:** Anja T. Fuglsang, atf@plen.ku.dk

# Supplementary Figures and Tables

## Supplementary table 1

**Primers and plasmids used in this study:**

| **Primer** | **5'-3' Sequence** | **Protein** | **Mutation** |
| --- | --- | --- | --- |
| Oli2153 | CGTCTCTTCTTCTTCTTCAGGTTC | kPSY1R | - |
| Oli2304 | CACCATGGTGCTTTCTAAAAGGAGAGTAAATCC | kPSY1R | - |
| Oli4119 | CTCGAGTTATCTTGGACCCGAGGG | kBAK1 | - |
| Oli4120 | CACCGCTTGGTGGCGAAGGAAA | kBAK1 | - |
| Oli4161 | GTACTTTAGTGGCCGTTGCGAGGCTAAAAGAGGAGCG | BAK1 | K317A |
| Oli4162 | CGCTCCTCTTTTAGCCTCGCAACGGCCACTAAAGTAC | BAK1 | K317A |
| Oli4185 | ACGAAACTGGCGGTTGCGAAACTCACAGGAGAC | PSY1R | K831A |
| Oli4186 | GTCTCCTGTGAGTTTCGCAACCGCCAGTTTCGT | PSY1R | K831A |
| Oli4253 | CACCATGGAGTCGAGTTATGTGG | SERK1 | - |
| Oli4254 | CCATGGCCTTGGACCAGATAACTCA | SERK1 | - |
| Oli4255 | CACCATGGGGAGAAAAAAGTTTGAA | SERK2 | - |
| Oli4256 | CCATGGTCTTGGACCAGACAACTC | SERK2 | - |
| Oli4257 | CACCATGACAAGTTCAAAAATGGAA | SERK4 | - |
| Oli4258 | CCATGGTCTTGGACCCGAGGGGT | SERK4 | - |
| Oli4259 | CACCATGGAACATGGATCATCCC | SERK5 | - |
| Oli4260 | CCATGGTCTTGGCCCCGAGGGGT | SERK5 | - |
| Oli4323 | CACCGGTCGACTCGCTTGGTGGCGACGAAG | kSERK1 | - |
| Oli4324 | GCGGCCGCTTACCTTGGACCAGATAACTC | kSERK1 | - |
| Oli4325 | CACCGGTCGACTCGCTTGGTGGCGTAGAAG | kSERK2 | - |
| Oli4326 | GCGGCCGCTTATCTTGGACCAGACAACTC | kSERK2 | - |
| Oli4327 | CACCGGTCGACTCGCTTGGTGGCTCAGAAG | kSERK4 | - |
| Oli4328 | GCGGCCGCTTATCTTGGACCCGAGGGG | kSERK4 | - |
| Oli4329 | CACCGGTCGACTCGCTTGGTGGCTGAGAAG | kSERK5 | - |
| Oli4330 | GCGGCCGCTTATCTTGGCCCCGAGGGG | kSERK5 | - |
| Oli4384 | GATAATGGAGCCAAACTGGCG | PSY1R | T826A |
| Oli4385 | CGCCAGTTTGGCTCCATTATC | PSY1R | T826A |
| Oli4386 | TAAGAAACTCGCCGGAGACTATG | PSY1R | T834A |
| Oli4387 | CATAGTCTCCGGCGAGTTTCTTA | PSY1R | T834A |
| Oli4388 | TATTGCGTCCATGATGCCGCCCGGATACTAATTT | PSY1R | S870A |
| Oli4389 | AAATTAGTATCCGGGCGGCATCATGGACGCAATA | PSY1R | S870A |
| Oli4390 | CGAGACATCAAGTCCGCCAACATCCTTTTGGAT | PSY1R | S933A |
| Oli4391 | ATCCAAAAGGATGTTGGCGGACTTGATGTCTCG | PSY1R | S933A |
| Oli4392 | TTTCGGGTTGGCCAGATTGATCC | PSY1R | S951A |
| Oli4393 | GGATCAATCTGGCCAACCCGAAA | PSY1R | S951A |
| Oli4394 | TTCCATATCGCGCCCATGTAACAAC | PSY1R | T959A |
| Oli4395 | GTTGTTACATGGGCGCGATATGGAA | PSY1R | T959A |
| Oli4396 | CACCCATGTAGCCACTGAGCTAG | PSY1R | T962A |
| Oli4397 | CTAGCTCAGTGGCTACATGGGTG | PSY1R | T962A |
| Oli4398 | CCATGTAACAGCCGAGCTAGTGG | PSY1R | T963A |
| Oli4399 | CCACTAGCTCGGCTGTTACATGG | PSY1R | T963A |
| Oli4400 | GCTAGTGGGCGCCTTGGGTTACA | PSY1R | T968A |
| Oli4401 | TGTAACCCAAGGCGCCCACTAGC | PSY1R | T968A |
| Oli4402 | ATGGGTGCATGCCATGAAAAGAG | PSY1R | T1022A |
| Oli4484 | CTCTTTTCATGGCATGCACCCAT | PSY1R | T1022A |
| Oli4485 | CATTGCTGAGAGAAGCCGGAAATGAAGAAGCGATG | PSY1R | S1040A |
| Oli4486 | CATCGCTTCTTCATTTCCGGCTTCTCTCAGCAATG | PSY1R | S1040A |
| Oli4487 | ATCGCGCCCATGTAACAGCCGAGCTAGTGGGCACA | PSY1R | T963A* |
| Oli4488 | TGTGCCCACTAGCTCGGCTGTTACATGGGCGCGAT | PSY1R | T963A* |
| Oli4491 | GAAGAAGAAGAGACGTAAAAGGGTGGGCGCGCCGACCCAG | PSY1R | STOP codon |
| Oli4492 | CTGGGTCGGCGCGCCCACCCTTTTACGTCTCTTCTTCTTC | PSY1R | STOP codon |

* These primers were used to generate the T963A mutation following the T959A mutation.
