# Supplementary material for: Activation of the LRR Receptor-Like Kinase PSY1R Requires Transphosphorylation of Residues in the Activation Loop
Source: Front Plant Sci. 2017 Nov 27;8:2005. doi: 10.3389/fpls.2017.02005 (PMC5712095; doi:10.3389/fpls.2017.02005)
Supplement: Supplementary file 2 [file Table_2.DOCX]

Supplementary Material

Activation of the LRR Receptor-Like Kinase PSY1R requires transphosphorylation of residues in the activation loop

**Christian B. Oehlenschlæger^1^, Lotte B. A. Gersby^1^, Nagib Ahsan^2,3^, Jesper T. Pedersen^1^, Astrid Kristensen^1^, Tsvetelina V. Solakova^1^, Jay J. Thelen^2^, and Anja T. Fuglsang^1*^**

*** Correspondence:** Anja T. Fuglsang, atf@plen.ku.dk

# Supplementary Figures and Tables

## Supplementary table 2

**Plasmids used in this study:**

| **Plasmid** | **Construct** | **Reference** |
| --- | --- | --- |
| pMP900 | AHA2-Cterm98 in pGEX-4T-1 | Fuglsang *et al* (2014) |
| pMP1350 | kPSY1R in pGEX-4T-1 |  |
| pMP2254 | kPSY1R-pAS1-CYH2 |  |
| pMP2577 | kPSY1RΔ24 in pGEX-4T-1 |  |
| pMP2266 | pX-CCFP Gateway. Yields a c-terminal fusion of a c-terminal fragment of CFP (aa.s 155-239) to the protein of interest. |  |
| pMP2267 | pX-NYFP Gateway. Yields a c-terminal fusion of an n-terminal fragment of YFP (aa.s 1-172) to the protein of interest. |  |
| pMP2310 | PSY1R-cCFP in pMP2266 |  |
| pMP2315 | PSY1R-nYFP in pMP2267 |  |
| pMP2858 | PSY1RΔ24-cCFP in pMP2266 |  |
| pMP2859 | PSY1RΔ24-nYFP in pMP2267 |  |
| pMP2860 | PSY1R-K831A-cCFP in pMP2266 | This study |
| pMP2861 | PSY1R-K831A-nYFP in pMP2267 | This study |
| pMP4107 | BAK1-nYFP in pMP2267 | This study |
| pMP4108 | BAK1-cCFP in pMP2266 | This study |
| pMP4290 | kBAK1-K317A in pDEST17 | This study |
| pMP4384 | kPSY1RΔ10 in pGEX-4T-1 | This study |
| pMP4465 | kPSY1R-K831A in pDEST17 | This study |
| pMP4466 | kPSY1R in pDEST17 | This study |
| pMP4579 | kPSY1R in pDEST15 | This study |
| pMP4580 | kPSY1R-T826A in pDEST15 | This study |
| pMP4581 | kPSY1R-T834A in pDEST15 | This study |
| pMP4582 | kPSY1R-S870A in pDEST15 | This study |
| pMP4583 | kPSY1R-S933A in pDEST15 | This study |
| pMP4584 | kPSY1R-S951A in pDEST15 | This study |
| pMP4585 | kPSY1R-T9596A in pDEST15 | This study |
| pMP4586 | kPSY1R-T962A in pDEST15 | This study |
| pMP4587 | kPSY1R-T963A in pDEST15 | This study |
| pMP4588 | kPSY1R-T968A in pDEST15 | This study |
| pMP4589 | kPSY1R-T1022A in pDEST15 | This study |
| pMP4590 | kPSY1R-S1040A in pDEST15 | This study |
| pMP4591 | kPSY1R-K831A-S951A-T959A-T963A in pDEST17 | This study |
| pMP4610 | kSERK1 in pDEST15 | This study |
| pMP4611 | kSERK2 in pDEST15 | This study |
| pMP4612 | kSERK4 in pDEST15 | This study |
| pMP4613 | kSERK5 in pDEST15 | This study |
| pMP4622 | kBAK1 in pDEST15 | This study |
| pMP4627 | SERK1-cCFP in pMP2266 | This study |
| pMP4628 | SERK2-cCFP in pMP2266 | This study |
| pMP4629 | SERK4-cCFP in pMP2266 | This study |
| pMP4630 | SERK5-cCFP in pMP2266 | This study |
| pMP4631 | SERK1-nYFP in pMP2267 | This study |
| pMP4632 | SERK2-nYFP in pMP2267 | This study |
| pMP4633 | SERK4-nYFP in pMP2267 | This study |
| pMP4634 | SERK5-nYFP in pMP2267 | This study |
| pMP4657 | kPSY1R-K831A in pDEST15 | This study |
