# Supplementary material for: Activation of the LRR Receptor-Like Kinase PSY1R Requires Transphosphorylation of Residues in the Activation Loop
Source: Front Plant Sci. 2017 Nov 27;8:2005. doi: 10.3389/fpls.2017.02005 (PMC5712095; doi:10.3389/fpls.2017.02005)
Supplement: Supplementary file 4 [file Image_2.pdf]

Supplementary Material

Activation of the LRR Receptor-Like Kinase PSY1R requires transphosphorylation of residues in the activation loop

Christian B. Oehlenschlæger[1], Lotte B. A. Gersby[1], Nagib Ahsan[2,3], Jesper T. Pedersen[1], Astrid Kristensen[1], Tsvetelina V. Solakova[1], Jay J. Thelen[2], and Anja T. Fuglsang[1*]

*** Correspondence:** Anja T. Fuglsang, atf@plen.ku.dk

1 Supplementary Figures and Tables

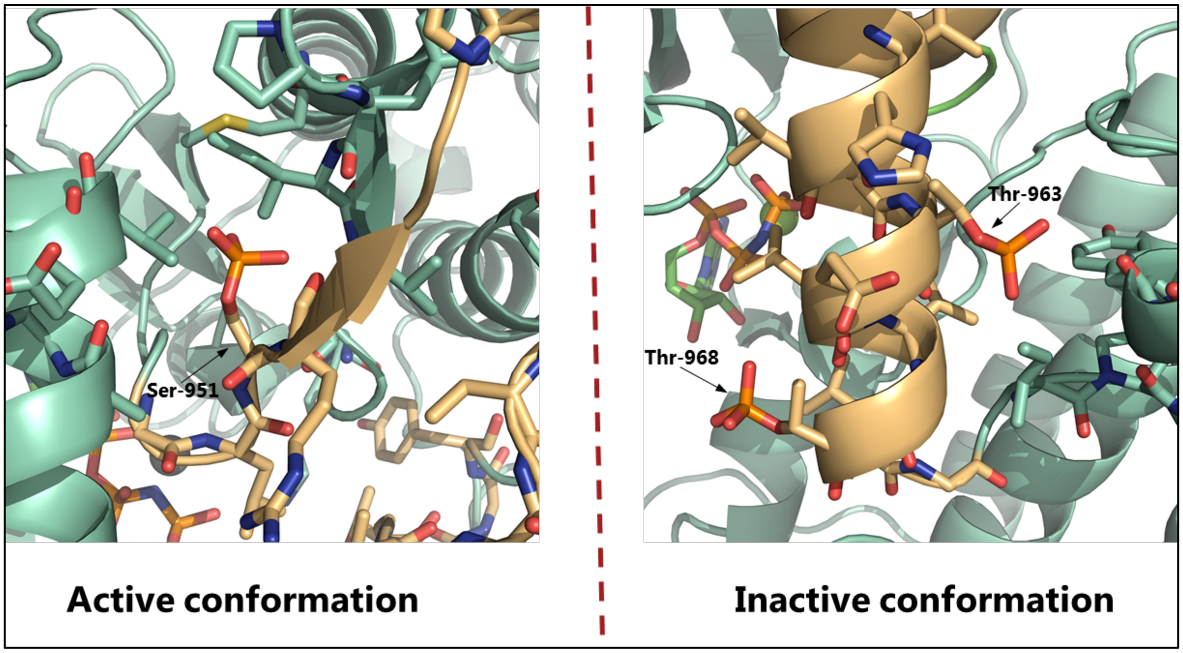

Supplementary figure 2

Homology model of the activation loop in active and inactive conformation.

The activation loop (light orange) has several phosphorylation sites. Phosphate residues have been modeled to Ser-951 in the active conformation and to Thr-963 and Thr-968 in the inactive kinase conformation. *Active conformation,* Phospho-Ser-951 is surrounded by hydrophobic residues in the active conformation that destabilize this state. *Inactive conformation,* Phosphor-Thr 963 and -968 does not readily show any interacting partners which might explain why the inactive state is unfavored by phosphorylation of especially Thr-968. The active site in all models is marked with the ATP analogue AMP-PNP and Mg^{2+} modelled from the template structures (PDB-id: 5LPV and 5UV4).
